# Genome Sequence Analysis of *Enterobacter* sp. C6, Found in the Pitcher Fluids of *Sarracenia rosea*

**DOI:** 10.1128/MRA.01214-19

**Published:** 2020-01-16

**Authors:** Shumeng Zhang, Srinivasa R. Chaluvadi, Jeffrey L. Bennetzen

**Affiliations:** a Institute of Bioinformatics, University of Georgia, Athens, Georgia, USA; b Department of Genetics, University of Georgia, Athens, Georgia, USA; Loyola University Chicago

## Abstract

An unclassified Enterobacter strain was isolated from the pitcher fluids of a Sarracenia rosea pitcher plant growing at Splinter Hill Bog in Alabama. Its genome was sequenced using the Illumina platform. A genome assembly of 4,673,815 bp was obtained. In total, 4,646 protein-encoding sequences and 72 RNA genes are predicted from this assembly.

## ANNOUNCEMENT

The genus *Enterobacter* was first proposed in 1960 (1). *Enterobacter* belongs to the Enterobacteriaceae, a group of Gram-negative, facultatively anaerobic, motile, and straight-rod bacteria (2). *Enterobacter* species are widespread in aquatic environments and are also present in the intestine of animals (3). *Enterobacter* species are sometimes found as pathogens that cause nosocomial infections such as urinary tract infections and bacteremia (4).

The strain used in this study was isolated from the fluids of *Sarracenia rosea* pitcher plants from Splinter Hill Bog (Alabama) in March of 2012. Pitcher fluids were plated onto medium used for the culture of Pyrococcus furiosus (5) and streaked from single colonies onto the same medium. An individual colony was then picked into nutrient broth for growth and eventual storage at −80°C in 25% glycerol. DNA was extracted using the Quick-DNA fungal/bacterial miniprep kit (catalog number D6005; Zymo Research). A paired-end library was prepared using the Nextera XT DNA library preparation kit v2 (catalog number FC-131-1002; Illumina) with an average insert size of ∼250 bp for Illumina MiSeq sequencing. Out of 14,784,154 Illumina raw reads, we retained 4,889,590 reads after quality filtering with Trimmomatic (version 0.36) (6). FastQC (version 0.11.4) was employed to evaluate the quality of the cleaned reads (7). SOAPdenovo2 (version r240; parameter -K 83) was used to assemble the reads into contigs (8). In total, 252 contigs (all >400 bp in length), with sequence coverage of more than 20×, were obtained. Both the NCBI Prokaryotic Genome Annotation Pipeline (9) and the online RAST server (RASTtk Toolkit) were used for annotation (10). Annotation by the NCBI Prokaryotic Genome Annotation Pipeline was provided with the GenBank submission. Default parameters were used for all software tools unless otherwise noted.

The final assembly has 4,673,815 bp. The GC content is ∼55.9%. The *N*_50_ and *L*_50_ values are 41,959 bp and 33, respectively. The sequence depth of the genome assembly is ∼260×. The genome contains a predicted 4,718 genes, which consisted of 4,646 protein-coding sequences and 72 RNA genes. About 38% of the protein-coding sequences (1,764 genes) for this genome were grouped in the functional subsystems of the RAST SEED server (10). Beta-lactam resistance gene *bla*_ACT-6_ and fosfomycin resistance gene *fosA* were predicted by the Center for Genomic Epidemiology (CGE)’s ResFinder (11). The strain was predicted to have a 75.5% probability of being a human pathogen according to CGE’s PathogenFinder (12). The two most closely related genomes, discovered by an NCBI BLASTn analysis using the nucleotide collection (nt/nr) database, are Enterobacter roggenkampii strain R11 (GenBank accession number NZ_CP019839) and Enterobacter cloacae subsp. *cloacae* ATCC 13047 (CP001918). The average nucleotide identities (ANI) of these two strains compared to *Enterobacter* sp. C6 are 93.7% and 87.9%, respectively (13). Several recent studies have demonstrated that a >95% ANI value is indicative of the same species (14). Both ANI values between *Enterobacter* sp. C6 and either E. cloacae strain are <95%, which suggests that *Enterobacter* sp. C6 is a new species of *Enterobacter*.

### Data availability.

This whole-genome shotgun project has been deposited in GenBank under accession number QPFW00000000. The raw read accession number is SRX6867760. The version described in this paper is the first version, QPFW01000000. The BioProject number is PRJNA476218, and the BioSample number is SAMN09428499.
